# In-vitro evaluation of *Indigofera heterantha* extracts for antibacterial, antifungal and anthelmintic activities

**DOI:** 10.1186/s40780-024-00328-y

**Published:** 2024-01-24

**Authors:** Showkat Ahmad Bhat, Mohammed Iqbal Zargar, Shahid Ud Din Wani, Ishfaq Mohiuddin, Mubashir Hussain Masoodi, Faiyaz Shakeel, Mohammad Ali, Seema Mehdi

**Affiliations:** 1 Department of Pharmaceutical Sciences, School of Applied Science and Technology, University of Kashmir, Srinagar, 190006 India; 2 Department of Zoology, Annamalai University, Annamalainagar, Tamil Nadu 608002 India; 3 Department of Pharmaceutics, College of Pharmacy, King Saud University, Riyadh, 11451 Saudi Arabia; 4 Department of Pharmacy Practice, East Point College of Pharmacy, Bangalore, 560049 India; 5 Present Address: Department of Pharmacology, Sri Adichunchanagiri College of Pharmacy, Adichunchanagiri University, B.G Nagar, Nagamagala, Bellur, Karnataka 571418 India; 6 Department of Pharmacology, JSS College of Pharmacy, Mysuru, 570015 India

**Keywords:** Microbial resistance, Bioactive compounds, *Indigofera Heterantha*, Antibacterial activity, Antifungal activity, Antihelmintic activity

## Abstract

**Background:**

Multidrug-resistant bacterial strains cause several serious infections that can be fatal, such as *Enterococcus faecium*, *Staphylococcus aureus*, *Klebsiella pneumonia*, *Acinetobacter baumannii*, *Pseudomonas aeruginosa*, and *Enterobacteriaceae* (often referred to as ESKAPE pathogens). Since ancient times, several indigenous medical systems in India have utilized diverse medicinal plants (approximately 80,000 species) as conventional treatments for a variety of illnesses. A member of the Fabaceae family, also referred to as “Himalayan indigo,” *Indigofera heterantha Wall*, is well known for its therapeutic properties.

**Methods:**

The present study investigated the antibacterial, antifungal and antihelmintic properties of the roots, bark, leaves, and flowers of *I. heterantha* from the Kashmir Himalayas. The effectiveness of the extracts against bacteria, fungi, and earthworms. Three of the tested organisms for bacteria were ESKAPE pathogens, as they are responsible for creating fatal bacterial infections. The antifungal potency of *I. heterantha* aqueous and methanolic extracts was evaluated using the Agar Well Diffusion Assay. The antihelmintic activity was carried out on an adult *Pheretima posthuma* Indian earth worm, which shares physiological and anatomical similarities with human intestinal roundworm parasites.

**Results:**

The methanolic extracts of root and bark have shown prominent activity against all bacterial strains, whereas aqueous extracts of flower, root, and leaves have shown promising activity against *Staphylococcus aureus*. The aqueous extract demonstrated good activity against *S. cerevisiae* at a concentration of 200 mg/ml with a zone of inhibition of 16 mm, while the methanolic extract displayed comparable activity against the fungal strains. The remaining two strains, *P. crysogenum* and *A. fumigatus*, were only moderately active in response to the extracts. All the extracts have shown anthelmintic activity except aqueous flower.

**Conclusion:**

These results will pave the way for the bioassay-guided isolation of bioactive constituents that may act as hits for further development as potential antibacterial agents against drug-resistant microbial and helminthic infections.

**Supplementary Information:**

The online version contains supplementary material available at 10.1186/s40780-024-00328-y.

## Introduction

 Multidrug-resistant bacterial strains cause a number of serious infections that can be fatal, such as *Enterococcus faecium*, *Staphylococcus aureus*, *Klebsiella pneumonia*, *Acinetobacter baumannii*, *Pseudomonas aeruginosa*, and *Enterobacteriaceae* (often referred to as ESKAPE pathogens). Antibiotics that are now available cannot be used to treat these infections. By 2050, if nothing is done, this will surpass cancer as the top cause of mortality [1]. Even in developed countries such as the US, such illnesses caused by drug-resistant microbes result in 23,000 fatalities each year. Similar conditions occur in Europe, but in the developing nations of Asia, such as Latin America, Africa, and India, they are considerably worse [2]. More concerningly, the pipeline for developing new antibiotic drugs has dried up, and the number of new antibiotics receiving clinical approval is falling. Finding new lead compounds with inventive modes of action that can counteract antimicrobial resistance is thus urgently needed. Additionally, natural products can be used to rationally design new lead compounds with enhanced potency against ESKAPE infections. Natural substances and medicinal plants have long been regarded as a primary source of medications to help humans [3, 4]. They are essential in the prevention of sickness and the treatment of different illnesses worldwide [5]. Alkaloids, flavonoids, glycosides, lignans, monoterpenes, lipids (phytosterols, tocopherols, saturated and unsaturated fatty acids), and vitamins are among the many pharmacologically active components they are thought to contain [6]. Therefore, a significant portion of recommended medications—nearly 25%—come from medicinal plants [7]. For almost 80% of people in underdeveloped countries, the World Health Organization (WHO) views the traditional homoeopathic system as a basic kind of treatment [8]. The therapeutic and curative properties of medicinal plants have been attributed to a variety of phytoconstituents [9]. Since ancient times, several indigenous medical systems in India have utilized diverse medicinal plants (approximately 80,000 species) as conventional treatments for a variety of illnesses [10]. From medicinal plants used to make prescription medicines, over 25% of active principles have been discovered [11].

A member of the *Fabaceae* family, also referred to as “Himalayan indigo,” *Indigofera heterantha Wall*, is well known for its therapeutic properties. *I. heterantha* leaves are crushed, and the resulting extract is used to treat disorders that affect the internal organs [12]. Internal wounds, throat infections, diabetes, toothaches, and jaw swellings are all treated using the plant’s bark and leaves [13]. Hepatitis is managed using the entire plant [14]. As a vermifuge, branches and leaf extracts are employed [15]. Abdominal pain can be relieved by rhizome bark powder [16]. *I. heterantha* has also been noted to exhibit antiulcerogenic, antioxidant, and antibacterial properties [17].

*Indigofera* genus is a rich source of bioactive compounds such as monoterpenes, triterpenes, steroids, lignins, tannins and alkaloids (Ghais uddin et al. 2011) [18]. Follwing compounds have been isolated from various species of the genus. For E.g. (Kaempferitrin C_27_H_30_O_14_*I. arrecta*) [19], Indigoidin C_10_H_8_N_4_O_4_*I. pseudomonas* [20], Indigotin, C_16_H_10_N_2_O_2_*I. tinctoria* [21] (Singh B et al., 2006). While some reported compound based on different geographical terrian as compared to our study have been extracted from seed of *I. heterantha* following compounds have been isolated and there structures have been obtained by mass spectrometry, Carbon 13 NMR and Proton NMR. Indigoferamide (S)-2-hydroxyN-((2S,3S,4R,E)-1,3,4-trihydroxyicos-16-en-2-yl)tricosanamide [22]. Indigoferate (Propyldotriacontanoate-C_35_H_70_O_2_), the compound has been obtained as white amorphous powder and has been found as moderate antibacterial agent. Indigoferone, 7-hydroxy-3-(3-methoxyphenyl)-4H-chromen-4-one (C_16_H_12_O_4_), and has been found to have carbonic anhydrase inhibiting activity. Other compounds reported are Dotriacontanoic acid, Formononetin, Quercetin, Quercetin 3-α-L-fucopyranoside, 3,5,7-Trihydroxy-6,4’-dimethoxyflavone, 4-Hydroxy-4-methyl-2-pentanone, and Two monoterpene glycosides [23].

The ether, ethyl acetate and methanol fractions of seed extract of *I. heterantha* had shown anti-fungal activity aganist *Microsporum canis* and *Fusarium Solani* and hydromethanolic root extract *of I. heterantha* shown significant activity aganist HSV-2 both in-vitro and in-vivo done in Balb mice [22, 24]. Three new phloroglucinol type compounds Indigoferin-A, Indigoferin-B and Indigoferin-C, along with a known compound β-sitosterol were isolated from the Indigofera gerardiana Syn; *I. heterantha* Wall. The compounds show significant Urease inhibitory activity [25]. Ethyl acetate soluble fraction of the methanolic extract of the whole plant of *I. heterantha* have been subjected to column chromatography, three compounds (one lignin and two acylphloroglucinols) were isolated which had shown lipoxygenase inhibiting activity (Aziz Ur Rehman et al., 2005) [26].

The present study investigated the antibacterial, antifungal properties and anti-helmintic properties of the roots, bark, leaves, and flowers of *Indigofera heterantha* from the Kashmir Himalayas. The effectiveness of the extracts against bacteria was assessed in *Pseudomonas aeruginosa* (MTCC1688), *Escherichia coli* (MTCC739), *Klebsiella pneumonia* (MTCC432), *Salmonella typhi* (MTCC3224), *Proteus vulgaris* (MTCC 426), *Bacillus subtilis* (MTCC441) and *Staphylococcus aureus* (MTCC96). Three of the tested organisms are ESKAPE pathogens, as they are responsible for creating fatal bacterial infections.

## Materials and methods

### Collection and identification of plant material

*Indigofera heterantha* is a deciduous shrub growing to 2–3 m (7–10 ft) tall and broad, young branches are angular, with slightly spreading white medifixed symmetrically 2-branched trichomes; stems are greyish brown, terete, with rounded lenticels, and covered in appressed medifixed trichomes. Dry, sunny slopes between 1,500 and 3,000 m in elevation, frequently creating dense brush, and in forests [27, 28]. *I. heteroantha* is a rather resistant plant; while temperatures between − 5 and − 10 °C might stunt top development, the rootstock is more resilient and can withstand temperatures as low as -15 °C most of the time [29]. Plants grow best in hot seasons with abundant sunshine, not flowering so freely in cool climates [30].

The *I. heterantha* employed in the study was obtained in Kashmir, India’s Gulmarg region of the Baramulla district during the month of June-July. Dr. Akhter H. Malik, a taxonomist with the Department of Botany at the University of Kashmir, recognized the plant at the Centre of Biodiversity and Taxonomy. A specimen of the plant was kept at the herbarium of the University of Kashmir’s Department of Botany in Srinagar under accession number 2325-KASH Herbarium. Roots, leaves, bark, and flowers were separated from the whole plant. For a period of two weeks, the plant material was dried in the shade. Dried roots, leaves, and bark were crushed into coarse powder by an electric grinder.

### Experimental

All chemicals used for phytochemical screening were obtained from CDH chemicals Ltd. Mumbai and were of analytical reagents (AR) grade and reagents were obtained from Medsource Ozone Biomedicals Pvt. Ltd. Delhi. Antibacterial activity was performed under laminar flow cabinet. The reagents used for qualitative and quantitative phytochemical estimation of the plant were obtained from Himedia company. Antioxidant activity was evaluated via UV visible spectrophotometer.

### Chemicals and Instruments

All chemicals were of highest purity (≥ 99.0%) and analytical grade. The chemicals used during the study includes Pet. ether, Methanol, Ethyl acetate, Acetic acid, AgNO3, Ammonia, AlCl_3_, CuSO_4_, Copper acetate, Diethyl ether, Ethanol, FeCl_3_, FeSO_4_, H_2_SO_4_, HCl, HNO_3_, Lead acetate, Magnesium metal strips, Mercury, Methanol, Na_2_CO_3_, NaCl, NaOH, n-butanol, Ninhydrin, Paraffin, Phloroglucinol, Picric acid, Hagers reagent, Wagners reagent, Dragendroffs reagent, Potassium dichromate, Potassium thiocyanate, Pyridine, Resorcinol, Sodium citrate, Sodium nitroprusside, TCA, Griess reagent, Potassium acetate, Glucose, Phenol, Gallic acid, AlCl3, Folin Ciocalteu reagent, Mueller Hinton agar (MHA), SDA, Fluconazole discs, Streptomycin discs, Xylene, 2,2-diphenyl-1-picrylhydrazyl (DPPH), Ascorbic acid.

The instruments used during the study include Incubator cum shaker, Hot air oven, Autoclave, Centrifuge, Desiccator, Digital weighing balance, Grinding mill, Oven, Rotary evaporator, UV-spectrometer, Laminar flow cabinet, Inoculating wire, Cork borer, Water bath, and general glass ware.

### Extraction yield

The cold extraction method was used to remove the plant material. Methanol and distilled water were the solvents employed in the cold extraction process. A 5000 ml macerating flask containing an accurately weighed 600 g of dried powdered leaves was filled with a 1:4 ratio of extracting solvent, and the mixture was allowed to stand at room temperature for 48 h while being constantly stirred. Once the soluble matter had dissolved, the mixture was strained, the marc was pressed, and the combing liquids were clarified by filtering. With the use of a rotary evaporator operating at low pressure, the extract was concentrated, and the solid extract was kept in a refrigerator for later use. A similar procedure was used to extract leaves aqueously. However, 300 g of powder were taken in a 2000 ml macerating flask. After adding a 1:4 ratio of extracting solvent to a precisely weighed 600 g of dried Root powder, the contents of the 5000 ml macerating flask were left to stand at room temperature for 48 h while being constantly stirred. Once the soluble matter had dissolved, the mixture was strained, the marc was pressed, and the combing liquids were clarified by filtering. With the aid of a rotary evaporator operating at lower pressure, the extract was concentrated, and the solid extract was then kept in a refrigerator for later use. A similar procedure was used to extract Root aqueously. nonetheless, 300 g of powder were consumed in a 2000 ml macerating flask. A precise weight of 300 g of dry Bark powder was weighed and placed in a 2000 ml macerating flask. After adding an extracting solvent in a 1:4 ratio and agitating the contents of the flask frequently for 48 h at room temperature, the soluble matter was dissolved. The combination was then strained, the marc was pressed, and the combing liquids were clarified by filtering. With the use of a rotary evaporator operating at low pressure, the extract was concentrated, and the solid extract was kept in a refrigerator for later use. A similar procedure was used to extract Bark aqueously. However, 200 g of powder was taken in a 2000 ml macerating flask. 200 g of dried flower powder, precisely weighed, was placed in a 2000 ml macerating flask. After adding an extracting solvent in a 1:4 ratio and agitating the contents of the flask frequently for 48 h at room temperature, the soluble matter was dissolved. The combination was then strained, the marc was pressed, and the combing liquids were clarified by filtering. With the use of a rotary evaporator operating at low pressure, the extract was concentrated, and the solid extract was kept in a refrigerator for later use. A similar procedure was used to extract flowers using water. However, 50 gms of the powder was taken in a 1000 ml macerating flask. Using a cold extraction technique using an aqueous and methanolic solvent system, crude extracts of several *I. heterantha* components were made [31].

### Qualitative phytochemical tests

Following the standard procedures described in the literature [32–34], the methanolic extracts of *I. heterantha* leaves, bark, roots, and flowers were subjected to qualitative analysis for secondary metabolites such as alkaloids, tannins, flavonoids, cardiac steroidal glycosides, proteins and amino acids, and carbohydrates.

### Infrared analysis

Likewise, infrared (IR) spectra were captured using a Perkin Elmer Spectrum 2 MIR Spectrometer (L1600235). The IR spectra (KBr pellets) between 400 and 4000 cm^-1^ were recorded using a total of 10 images [35]. We recorded the FTIR spectra to search for potential functional groups [36–38].

### Antibacterial activity

The bacterial strains used to evaluate the in vitro antibiotic activity of I. heterantha crude extracts were purchased from the Microbial Type Culture Collection (MTCC) at Chandigarh, India’s Institute of Microbial Technology (IMTECH). *Escherichia coli* (MTCC 739), *Proteus vulgaris* (MTCC 426), *Staphylococcus aureus* (MTCC 96), *Pseudomonas aeruginosa* (MTCC1688), *Bacillus subtilis* (MTCC441), and *Klebsiella pneumoniae* (MTCC432) are some of the species that are included. Both aqueous and methanolic extracts had their antibacterial activity evaluated using the Agar Well Diffusion Method. The Mueller Hinton Agar Well Diffusion Method was modified somewhat for use in the susceptibility tests. The bacterial strains were adjusted to a turbidity of 0.5 Mac Farland norm (108 CFU/ml) and suspended in sterile water after growing on nutritional agar for 18 h at 37 °C. The turbidity was measured using a UV spectrophotometer at 625 nm. Mueller Hinton Agar (MHA) medium was prepared using tap water and sterilized in an autoclave at 121 °C and 16 psi for 14 min in the Agar Well Diffusion Process. After sterilization, the medium was added to clean Petri plates in the laminar hood, where the plates were left to harden. Using a sterile cork borer with a 5 mm diameter, wells were created in each Petri plate that were uniform and equally spaced. On the plates, a standardized inoculum was used to inoculate each test bacteria (0.1 ml) of (0.5 McFarland) and spread with a spreader. Plant extracts were loaded into various peripheral wells at different concentrations (10 mg/ml, 30 mg/ml, 50 mg/ml, 80 mg/ml, and 100 mg/ml). Each Petri plate had a positive control of gentamycin (10 mcg/disc), whereas a different Petri plate contained a negative control of 10% dimethyl sulfoxide (DMSO). For 18 to 24 h, the Petri plates were incubated at 37 °C in an incubator. Zones of inhibition were then looked for on the plates. The inhibitory zone diameters, which are measured in millimeters (mm), were used to determine the antibacterial potential.

### Antifungal activity

To assess the in vitro antifungal activity of the fungal strains from the Microbial Type Culture Collection (MTCC), Chandigarh, India, crude methanolic and aqueous extracts of *I. heterantha* were utilized. These strains include *Saccharomyces cerevisiae* (MTCC 170), *Candida albicans* (MTCC 227), *Penicillium chrysogenum* (MTCC 947), and *Aspergillus fumigatus* (MTCC 426). The antifungal potency of *I. heterantha* aqueous and methanolic extracts was evaluated using the Agar Well Diffusion Assay. Each test fungus was injected with 0.1 ml of standardized inoculum (0.5 McFarland), homogenized, and then placed into a sterile Petri plate at a constant depth of 4 mm on sterile molten Sabouraud dextrose agar media. The Petri plates were able to harden in the laminar hood. Using a sterile cork borer with a diameter of 6 mm, five wells were created on the periphery and one well in the center of each Petri plate. Plant extract in five different peripheral wells at concentrations of 10 mg/ml, 30 mg/ml, 50 mg/ml, 80 mg/ml, and 100 mg/ml totaled 40 l. A separate Petri plate had one well with nystatin (10 mg/ml) and fluconazole (25 mg/ml) inserted as a disc. In a different well, 10% dimethyl sulfoxide was used as a negative control. The plates were stored at 32 °C for 24 to 36 h. After incubation, the plates were examined for areas of inhibition. The antifungal potential was measured by measuring the widths of the inhibitory zones in millimeters (mm) using a standard measurement scale.

### Anthelmintic activity

#### Experimental worms

Because the adult Indian earthworms (*Pheretima posthuma*) share structural similarities with human intestinal roundworm parasites, these investigations were conducted on them exclusively. After being removed from the damp earth, they had a water wash to get rid of all the excrement.

#### Administration of albendazole

Using 0.5% w/v of CMC as a suspending agent, albendazole (20 mg/ml) was produced and administered in accordance with the extract technique.

#### Administration of extract

Using 0.5% w/v of CMC as a suspending agent, the suspension of methanolic and aqueous extracts of *I. hethrantha* was created in different concentrations (50, 100 mg/ml). The final volume was increased to 10 ml for each concentration. The standard treatment was albendazole. Two earthworms per group, in groups of roughly equal size, were introduced into each 10 millilitre of the required medication and extract concentration in the petridish.

#### Experimental design

 The antihelmintic activity was carried out in compliance with Partap et al., 2012 [39]’s methodology on an adult *Pheretima posthuma* Indian earth worm, which shares physiological and anatomical similarities with human intestinal roundworm parasites. *Pheretima posthuma* were put in a petridish with two distinct quantities of ethanolic and aqueous extract from various *I. heterantha* Sects. (50 & 100 mg/ml). Two worms were added to each petridish, and the worms’ paralysis or death was monitored. After it was determined that worms did not move in response to external stimuli or when they were shook violently, the mean time for paralysis was recorded, which was the point at which no movement of any kind could be seen. The time of worm death (min) was also recorded. The test findings were contrasted with samples treated with the reference chemical albendazole (20 mg/ml).

## Results

### Yield of plant extract

The extraction process used was cold extraction and solvent used where methanol and water. Aqueous leaves has shown higher percentage yield 16.77% followed by aqueous root 15.33% methanolic leaves 13.64% methanolic root 12.73% aqueous bark 12.66% methanolic bark 10.41% aqueous flower 9.22% methanolic flower 7.48%. From the above it may be conclude that solvent water is showing higher percentage yield than the methnol (Table 1).

**Table 1:** Extraction of different parts of *I. heterantha* by using the methanolic and aqueous solvent system, with their percentage extraction yields^a^

Indigofera Heterantha (Part)	The solvent system used for extraction	Percentage extraction yield
Leaves	Methanol Water	13.64% 16.77%
Roots	Methanol Water	12.73% 15.33%
Bark	Methanol Water	10.41% 12.66%
Flower	Methanol Water	7.48% 9.22%

^a^Percentage extraction yields were higher in the aqueous solvent system rather than the methanolic solvent system

### Phytochemical screening

Several secondary metabolites were found in the I. hethrantha extract during phytochemical screening; they are mentioned in Table 2. The presence of the phytoconstituents under investigation, including alkaloids, glycosides, tannins, flavonoids, and saponins, was detected by phytochemical screening. However, the findings of certain tests were inconsistent. The Legal Test, showed no Glycosides in all extract, but the Keller Killiani Test did. Similarly, the FeCl_3_ Test revealed no presence of Alkaloids, but Wagner’s Test did.

**Table 2:** Qualitative phytochemical assessment

Class	Test	Root		Bark		Leaves		Flower	
		Methanolic	Aqueous	Methanolic	Aqueous	Methanolic	Aqueous	Methanolic	Aqueous
**Alkaloids**	Wagner’s Test	+	+	+	+	+	+	+	+
	FeCl_3_ Test			+	+	+	+		
**Glycosides**	Legal Test								
	Keller Killiani Test	+	+	+	+	+	+		
**Tannins**	Alkaline Reagent Test	+	+	+	+	+	+		
	Lead Acetate Test	+	+	+	+	+	+	+	+
**Flavonoids**	Alkaline Reagent Test	+	+	+	+	+	+		
	FeCl_3_ Test			+	+	+	+		
**Saponins**	Lead Acetate Test	+	+	+	+	+	+	+	+
	Saponin Frothing Test	+	+	+	+	+	+	+	+

There were numerous phytoconstituents present in all *I. heterantha* extracts (leaf, root, and bark), including carbohydrates, phenols, alkaloids, flavonoids, glycosides, tannins, terpenoids, and others.

### Infrared analysis

In the functional group region of the compound’s experimental IR spectra, the peaks for olefinic/aromatic C-H, aliphatic C-H, lactone C = O, and C-C stretching vibrations are particularly prominent. O-H, C-H, and C-O bending vibrations can be seen in the fingerprint region [40].

**Fig. 1:**
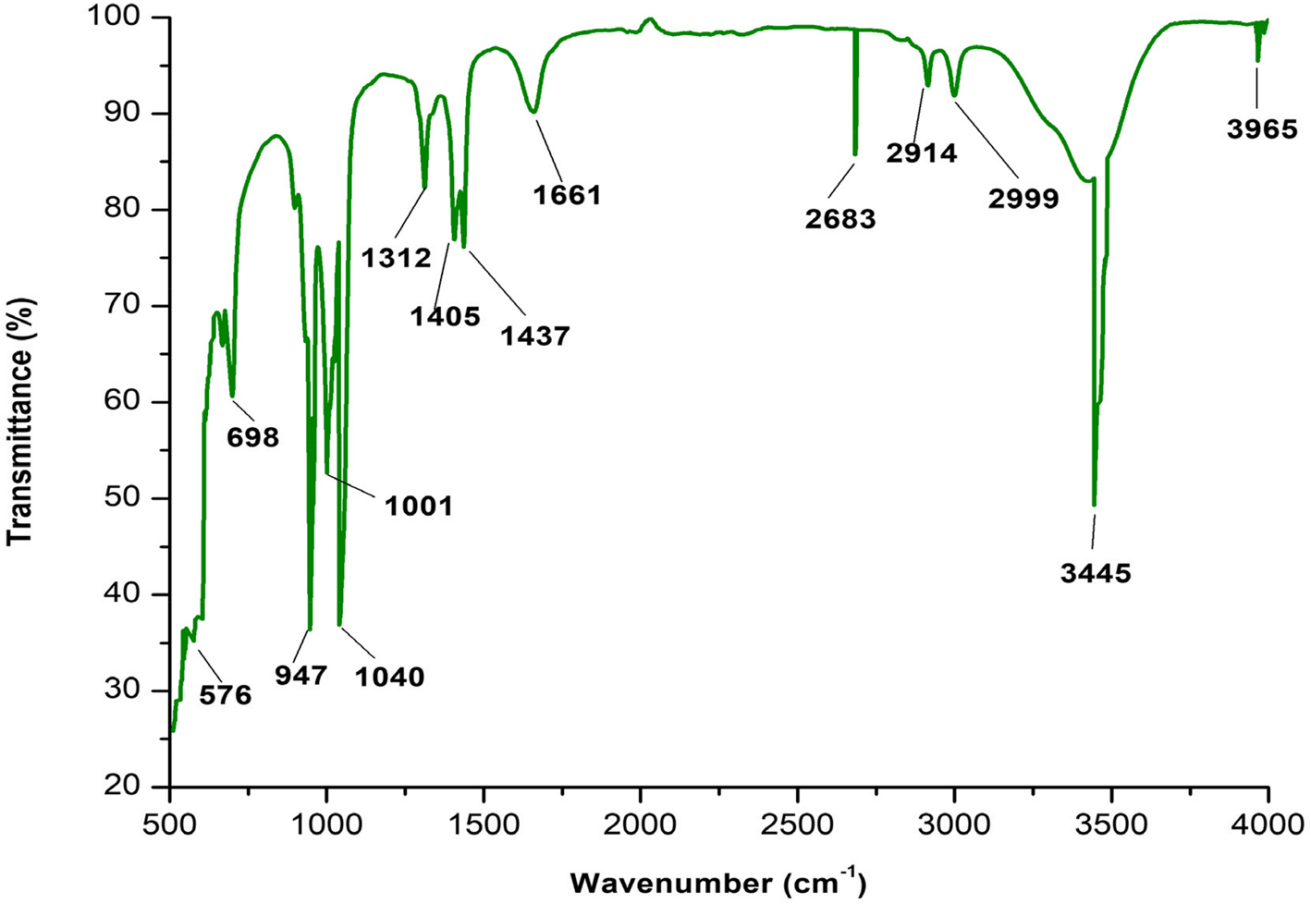
Prominent fundamental vibrations of compounds

The extracts from *I. heterantha* had functional groups, as evidenced by the FTIR spectrum, which had peaks at 3965 cm^-1^ (alcohols and amines), 3445 cm^-1^ (alcohols, amine & amides, and carboxylic acids), 2999 cm^-1^ (alcohols, amine salts, carboxylic acids, and alkanes), 2914 cm^-1^ (alcohols, amine salts, carboxylic acids, and alkanes), 2683 cm^-1^ (alkanes and carboxylic acid), 1661 cm^-1^ (alkene, aromatic compounds, imine, and am-ides), 1437 cm^-1^ (alkanes, nitro, and carboxylic acids), 1405 cm^-1^ (alkenes, nitro, carboxylic acids, and sulfate, sulfonyl chlorides), 1312 cm^-1^ (phenol, aromatic amines, sulfones, sulfonyl chlorides, sulfonate and sulfonamides), 1040 cm^-1^ (fluoro compounds, amines, sulfoxide anhydrides), 1001 cm^-1^ ( amines, fluorides), 947 cm^-1^ (alkenes), 698 cm^-1^ (aromatic compounds, alkenes, and alkyl halides) and 576 cm^-1^ Halo compounds (Fig. 1) Table 3.

**Table 3:** Prominent fundamental vibrations

S. NO	Wavenumber (cm^-1^)	Compound	Functional groups
1	3965	Alcohol	O-H stretching
		Amine	N-H stretching
2	3445	Alcohol	O-H stretching
		Amine and amides	N-H stretching
		Carboxylic acid	O-H stretching
3	2999	Alcohol	O-H stretching
		Amine salt	N-H stretching
		Alkane	C-H stretching
		Carboxylic acid	O-H stretching
4	2914	Alcohol	O-H stretching
		Carboxylic acid	O-H stretching
		Alkane	C-H stretching
		Amine salt	N-H stretching
5	2683	Alkane	C-H stretching
		Carboxylic acid	O-H stretching
6	1661	Alkene	C = C stretching
		Aromatic compound	C-H bending
		Imine/oxime	C = N stretching
		Amide	C = C bending
7	1437	Alkane	C-H bending
		Nitro	N = N bending
		Carboxylic acid	O-H bending
8	1405	Alkane	C-H bending
		Carboxylic acid	O-H bending
		Nitro	N = N bending
		Alcohol	O-H bending
		Sulfate, Sulfonyl chlorides	S = O stretching
9	1312	Phenol	O-H bending
		Aromatic Amine	C-N stretching
		Sulfones, Sulfonyl chlorides, Sulfates, Sulfonamides	S = O stretching
10	1040	Fluoro compound	C-F stretching
		Amine	C-N stretching
		Sulfoxide	S = O stretching
		Anhydride	CO-O-CO stretching
11	1001	Amine	C-N stretching
		Fluoride	C-F stretching
12	947	Alkene	C = C bending
13	698	Aromatic compounds	C-H bending
		Alkene	C-H bending
		Alkyl halides	C-Cl stretching
14	576	Halo compounds	C-X stretching

While the corresponding vibration in the experimental spectrum appears at 1661 cm^-1^, the C-H bending vibration is simulated at 1437 cm^-1^. At 1312 cm^-1^, the C-O stretching vibration is simulated. The fingerprint region frequently exhibits bending vibrations of 1040 (scissoring), 1040 (bending), 947 (ring breathing), and 698 cm^-1^ (wagging).

### In vitro antibacterial activity

The agar well diffusion method was used to assess the antibacterial activity in vitro using gentamycin as a positive control and DMSO as a negative control. *Escherichia coli* (MTCC443), *Proteus vulgaris* (MTCC426), *Staphylococcus aureus* (MTCC96), *Pseudomonas aeruginosa* (MTCC1688), *Bacillus subtilis* (MTCC441), and *Klebsiella pneumoniae* (MTCC432) were the six bacterial strains employed in the investigation.

**Fig. 2:**
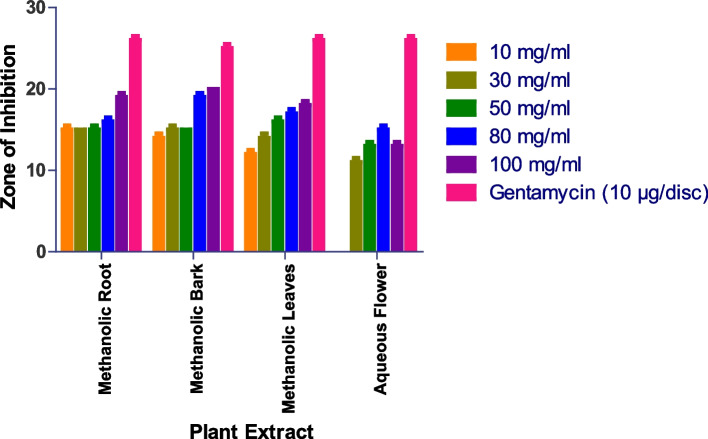
In vitro antibacterial action against *Bacillus subtilis.* Data are expressed as mean ± SD (*n* = 3, *p* < 0.0001)

At 100 mg/ml, methanolic bark extract inhibits *Bacillus subtilis* with a 20 mm zone of inhibition, followed by methanolic root with a 19 mm zone and aqueous root, aqueous bark, and aqueous leaves with no action (Figs. 2 and S2). While as Methanolic Flower, Aqueous Root and Aqueous Bark did not show any anti-bacterial activity.

**Fig. 3:**
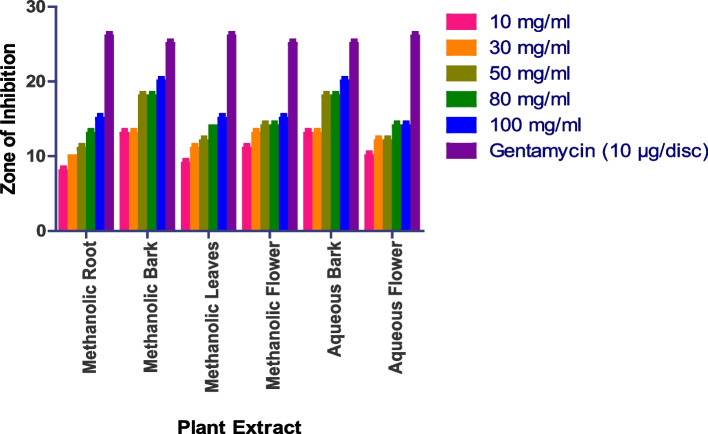
In vitro antibacterial action against *E. coli.* Data are expressed as mean ± SD (*n* = 3, *p* < 0.0001)

Methanolic bark extract inhibits *E. coli* by 20 mm diameter zone inhibition at 100 mg/ml and 13 mm diameter zone inhibition at 10 mg/ml. Methanolic flower extract shows a 15 mm zone at 100 mg/ml, methanolic root shows a 15 mm zone at 100 mg/ml while aqueous bark shows a 20 mm zone at 100 mg/ml aqueous flower shows a 14 mm zone at 100 mg/ml aqueous root does not show any activity (Figs. 3 and S3). While as, Aqueous Root and Aqueous leaves did not show any anti-bacterial activity.

**Fig. 4:**
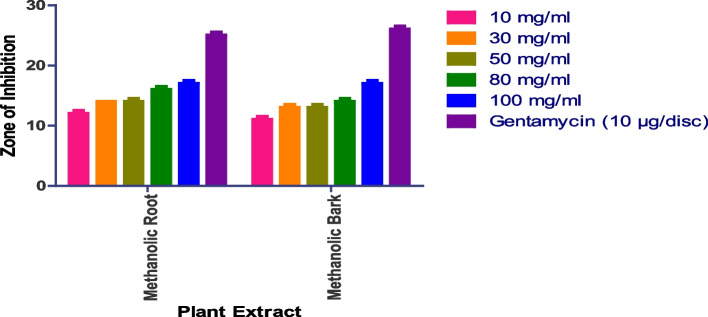
In vitro antibacterial action against *Proteus vulgaris.* Data are expressed as mean ± SD (*n* = 3, *p* < 0.0001)

The methanolic root extract of *Proteus vulgaris* displays a 17 mm inhibition zone at 100 mg/ml and a 12 mm inhibition zone at 10 mg/ml. At 100 mg/ml, methanolic bark displays a 17 mm inhibition zone and no activity at 10 mg/ml, while aqueous extracts display no activity (Figs. 4 and S4). While as, Methanolic Leaves, Methanolic Flower, Aqueous Root, Aqueous Bark, Aqueous Leaves, Aqueous Flower did not show any anti-bacterial activity.

**Fig. 5:**
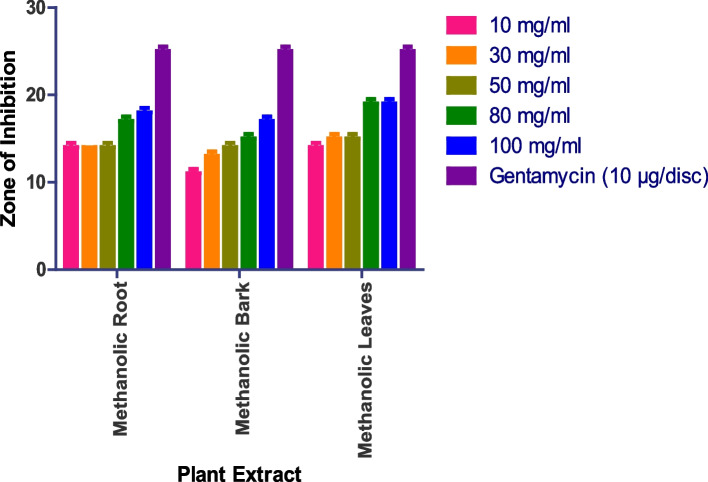
In vitro antibacterial action against *Pseudomonas aeruginosa.* Data are expressed as mean ± SD (*n* = 3, *p* < 0.0001)

The methanolic root extract inhibits *Pseudomonas aeruginosa* and revealed an 18 mm zone at 100 mg/ml and a 14 mm zone at 10 mg/ml. At 100 mg/ml, methanolic leaves displayed a 19 mm patch, while methanolic flower and aqueous extracts exhibited no activity (Figs. 5 and S5). Whereas as Methanolic Flower, Aqueous Root, Aqueous Bark, Aqueous Leaves, Aqueous Flower did not show any anti-bacterial activity.

**Fig. 6:**
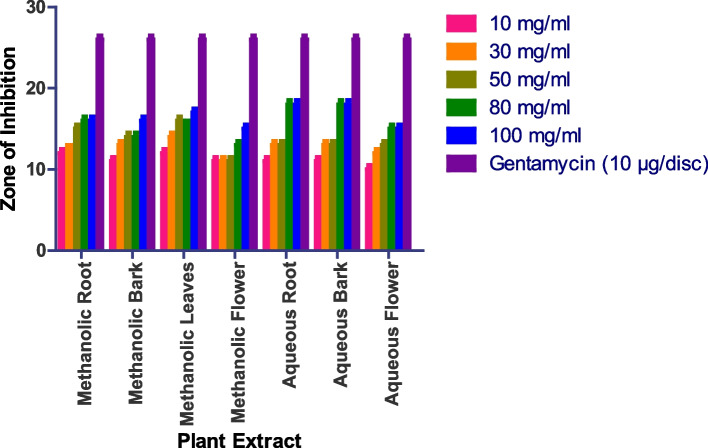
In vitro antibacterial action against *Staphylococcus aureus.* Data are expressed as mean ± SD (*n* = 3, *p* < 0.0001)

Methanolic root at 100 mg/ml showed a 16 mm zone, methanolic bark at 100 mg/ml showed a 12 mm zone, and methanolic bark at 10 mg/ml showed a 16 mm zone for *Staphylococcus aureus* (Figs. 6 and S6). Methanolic leaves had a 17 mm zone at 100 mg/ml and an 11 mm zone at 10 mg/ml. At 100 mg/ml, the aqueous root displays a 15 mm zone, while at 10 mg/ml, it shows a 12 mm zone. At 100 mg/ml, the aqueous bark had an 18 mm zone, the aqueous leaves had a 17 mm zone, and the aqueous flowers had a 15 mm zone. Whereas, Aqueous Flower did not show any anti-bacterial activity.

**Fig. 7:**
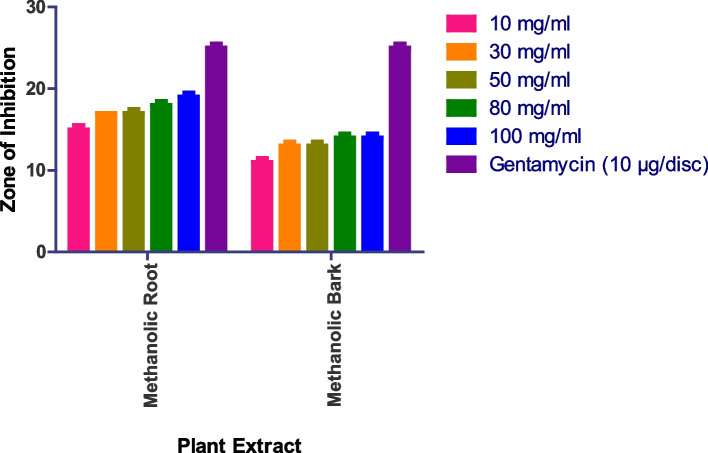
In vitro antibacterial action against *Klebsiella pneumoniae.* Data are expressed as mean ± SD (*n* = 3, *p* < 0.0001)

At 100 mg/ml, the methanolic root extract revealed a 19 mm zone for *Klebsiella pneumoniae*, and at 30 mg/ml, it showed a 17 mm zone. At 100 mg/ml, methanolic bark shows a 14 mm zone, while aqueous extracts display no activity (Figs. 7 and S7). Whereas as Methanolic Leaves and Flower, Aqueous Root, Aqueous Bark, Aqueous Leaves, Aqueous Flower did not show any anti-bacterial activity.

### In vitro antifungal activity

**Fig. 8:**
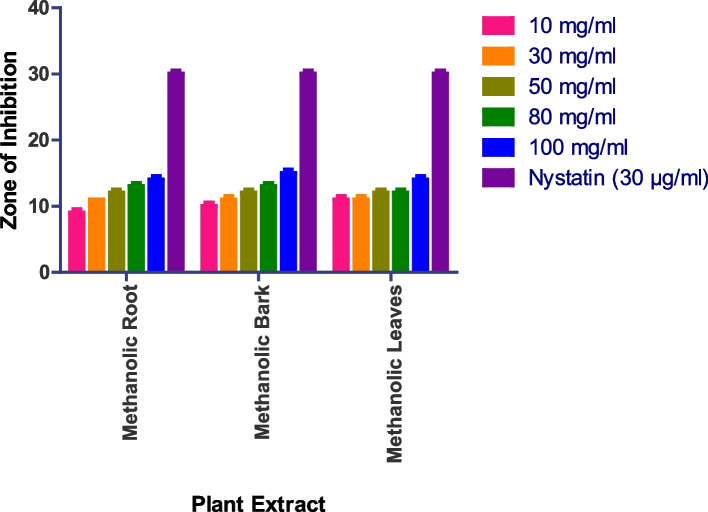
In vitro antibacterial action against *Penicillium chrysogenum.* Data are expressed as mean ± SD (*n* = 3, *p* < 0.0001)

The in vitro antifungal activity was assessed using the agar well diffusion method, with DMSO acting as a negative control and nystatin and fluconazole serving as positive controls. The antifungal activity of four fungal strains was tested: *Saccharomyces cerevisiae (MTCC170), Penicillium chrysogenum (MTCC947), Aspergillus fumigatus (MTCC 426), and Candida albicans (MTCC 227).* Methanolic roots, bark, and leaves show prominent activity, while methanolic flowers, aqueous roots, bark, leaves, and flowers do not show any activity. In the case of *Penicillium chrysogenum*, the methanolic bark shows a 15 mm zone at 100 mg/ml methanolic root shows a 14 mm zone at 100 mg/ml, while methanolic leaves show a 14 mm zone at 100 mg/ml (Figs. 8 and S8). Whereas as Methanolic Flower, Aqueous Root, Aqueous Bark, Aqueous Leaves, Aqueous Flower did not show any anti-fungal activity.

**Fig. 9:**
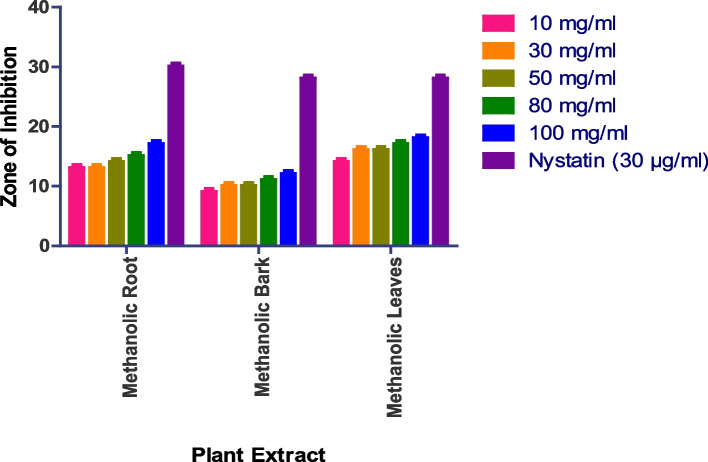
In vitro antibacterial action against *Aspergillus fumigatus.* Data are expressed as mean ± SD (*n* = 3, *p* < 0.0001)

The methanolic root extract showed a 17 mm zone of inhibition at 100 mg/ml, the methanolic leaf extract showed an 18 mm zone at 100 mg/ml, and the methanolic bark extract showed a 12 mm zone at 100 mg/ml against *Aspergillus fumigatus* (Fig. 9 and S9). Whereas as Methanolic Flower, Aqueous Root, Aqueous Bark, Aqueous Leaves, Aqueous Flower did not show any anti-fungal activity.

**Fig. 10:**
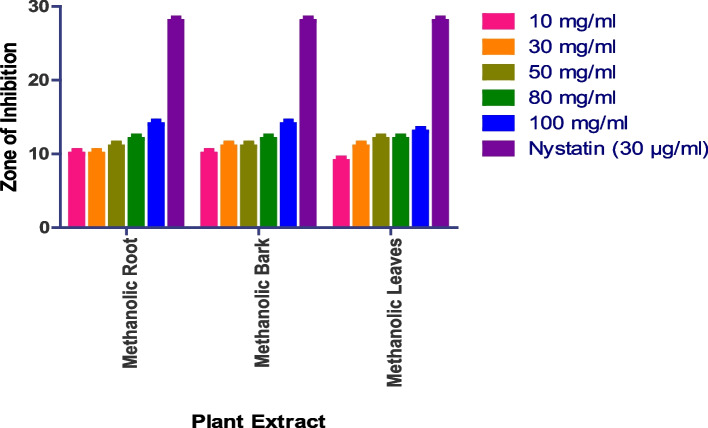
In vitro antibacterial action against *Saccharomyces cerevisiae.* Data are expressed as mean ± SD (*n* = 3, *p* < 0.0001)

The methanolic root extract showed a 14 mm zone at 100 mg/ml, the methanolic bark extract showed a 14 mm zone at 100 mg/ml, and the methanolic leaf extract showed a 13 mm zone at 100 mg/ml against *Saccharomyces cerevisiae* (Fig. 10 and S10). Whereas as Methanolic Flower, Aqueous Root, Aqueous Bark, Aqueous Leaves, Aqueous Flower did not show any anti-fungal activity.

**Fig. 11:**
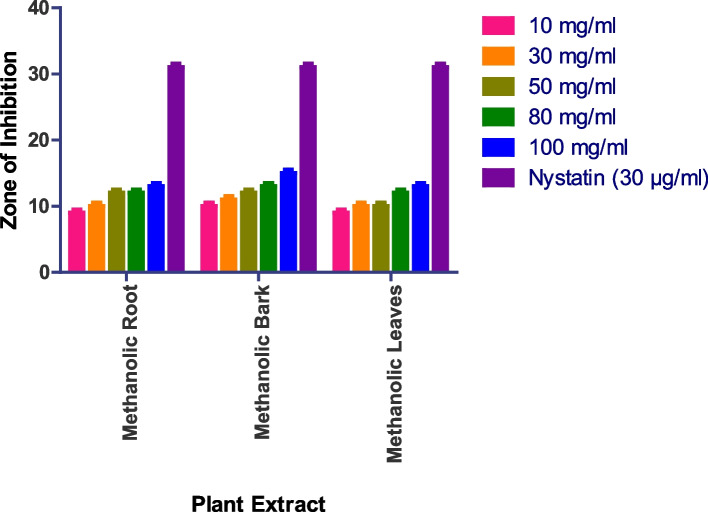
In vitro antibacterial action against *Candida albicans.* Data are expressed as mean ± SD (*n* = 3, *p* < 0.0001)

Similarly, the methanolic leaf extract showed a 13 mm zone at 100 mg/ml, the methanolic bark extract showed a 15 mm zone at 100 mg/ml, and the methanolic root extract showed a 13 mm zone at 100 mg/ml against *Candida albicans* (Fig. 11 and S11). Whereas as Methanolic Flower, Aqueous Root, Aqueous Bark, Aqueous Leaves, Aqueous Flower did not show any anti-fungal activity.

### Anthelmintic activity

**Fig. 12:**
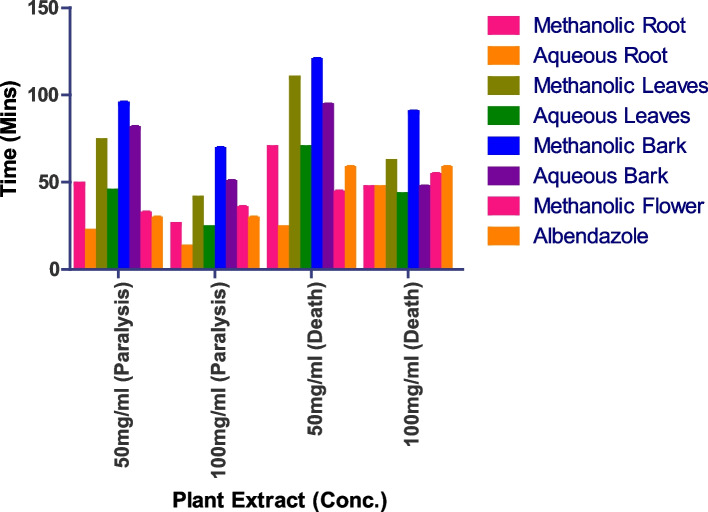
Anthelmintic activity of *I. heterantha* at different concentrations against *Pheretima posthuma.* Data are expressed as mean ± SD (*n* = 3, *p* < 0.0001)

Since the adult Indian earth worm *Pheretima posthuma* bears morphological and physiological similarities to human intestinal round worm parasites, the anthelmintic activity was conducted on this species. Aqueous root extract at 100 g/ml death occurs at 24 min and paralysis at 13 min at 50 mg/ml death occurs at 47 min and paralysis at 22 min. Methanolic flower extract at 100 mg/ml death occurs at 44 min and paralysis at 32 min at 50 mg/ml death occurs at 4 min and paralysis at 35 min; aqueous leaves extract at 100mgml death occurs at 43 min and paralysis at 24 min at 50 mg/ml death occurs at 70 min and paralysis at 45 min (Figs. 12 and S12).

Methanolic root at 100 g/ml death occurs at 47 min and paralysis at 26 min at 50 mg/ml death occurs at 70 min and paralysis at 59 min. Aqueous bark at 100 g/ml death occurs at 70 min and paralysis at 50 min at 50 g/ml death occurs at 94 min and paralysis at 81 min. Methanolic bark at 100 mg/ml death occurs at 90 min and paralysis at 69 min at 50 mg/ml death occurs at 120 min and paralysis at 95 min. Aqueous flower does not show any activity. Methanolic root, flower and aqueous leaves have shown prominent anti-helminthic activity.

## Discussion

The *I. heterantha* extracts used in the current study showed the existence of a range of bioactive components, including alkaloids, glycosides, tannins, flavonoids, & saponins. These phytochemicals could be responsible for the pharmacological properties. The compounds that reported antimicrobial activities of purified *I. heterantha* lectin obtained from seeds by Ion exchange chromatography and gel filtration chromatography technique has exhibited a significant antibacterial effect on four strains namely *Klebsiella pnuemoniae*, *Staphylococcus aureus*, *Escheri-chia coli*, and *Bacillus subtilis* [41]. The antibacterial activity of several *I. heterotrantha* extracts was evaluated in the current investigations against a variety of bacterial strains.

When measuring in vitro antibacterial activity using the agar well diffusion method, gentamycin was employed as a positive control, and DMSO was employed as a negative control. Two strains of *gram-positive* and two strains of *gram-negative* bacteria were tested for antibacterial activity using the methanolic and aqueous root extracts, and it was shown that they were all efficient. *Proteus vulgaris, Staphylococcus aureus, and Escherichia coli* all had their growth suppressed by the extracts. Maximum zones of inhibition for all four strains of methanolic extract ranged from 14.3 to 16.5 mm. With a 15.6 mm zone of inhibition, the aqueous extract demonstrated good effectiveness against *S. typhi* as well. Methanolic bark and root extract inhibited *Bacillus subtilis Proteus vulgaris*, and *Klebsiella pneumoniae*. Methanolic bark, flower, root, and aqueous bark extract inhibited *E. coli*. Methanolic root and leaves extract inhibited *Pseudomonas aeruginosa.* Methanolic root, leaves, bark, aqueous root and bark extract inhibited *Staphylococcus aureus*.

The antifungal activity of four fungal strains was tested: *Saccharomyces cerevisiae (MTCC170), Penicillium chrysogenum (MTCC947), Aspergillus fumigatus (MTCC 426), and Candida albicans (MTCC 227).* Methanolic roots, bark, and leaves show prominent activity, while methanolic flowers, aqueous roots, bark, leaves, and flowers do not show any activity. The aqueous extract demonstrated good activity against *S. cerevisiae* at a concentration of 200 mg/ml with a zone of inhibition of 16 mm, while the methanolic extract displayed comparable activity against the fungal strains. The remaining two strains, *P. crysogenum* and *A. fumigatus*, were only moderately active in response to the extracts.

Since the adult Indian earth worm *Pheretima posthuma* bears morphological and physiological similarities to human intestinal round worm parasites, the anthelmintic activity was conducted on this specie. Methanolic root, flower and aqueous leaves have shown prominent anti-helminthic activity. Aqueous flower does not show any activity. Methanolic flower extract at 100 mg/ml death occurs at 44 min and paralysis at 32 min at 50 mg/ml death occurs at 4 min and paralysis at 35 min. Methanolic root at 100 g/ml death occurs at 47 min and paralysis at 26 min at 50 mg/ml death occurs at 70 min and paralysis at 59 min.

Herpes simplex virus 2 has been tried in vitro and in vivo against the roots of *I. heterantha*. It was discovered that the hydromethanolic extract operated at several stages of viral entry and had a strong and targeted activity against the virus in vitro. When the extract was applied topically to HSV-2-infected mice, the clinical symptoms significantly decreased and the animal’s survival increased [24].

However, these investigations lack dose-effect data because only one dose of the extract has been examined in vivo. Many species of Indigofera, such as I. *heterantha*, have long been used to treat renal diseases [42–44].

The present study’s results are encouraging because the extracts exhibited strong antimicrobial activity in vitro and their mechanisms of action were investigated in vitro. However, there are still gaps in our expanded understanding of the genus *Indigofera*, necessitating more research. More research needs to be done on certain species due to their historical uses. Notably, *I. heterantha*—which is commonly used in Pakistan and India to treat pain and digestive issues—exemplifies this.

## Conclusions

The antimicrobial and antifungal activity of various extracts of *I. heterantha* has been reported. The in vitro antibacterial activity shows that roots, bark, leaves, and flowers possess antibacterial activity. Methanolic root and bark have prominent activity on all strains, while aqueous flower, root, and leaves show prominent activity on *Staphylococcus aureus. In vitro* methanolic root, bark, and leaves have prominent antifungal activity, whereas methanolic flower, aqueous root, bark, leaves, and flower do not possess any significant antifungal activity. The results will further guide us in the isolation of the desired phytoconstituents responsible for the antimicrobial and antifungal activity.

The anthelmintic, antibacterial, and antifungal potential of roots, leaves, bark, and flower extracts of *I. heterantha* of Kashmir region has not been evaluated till date, and this work presents the first report of the plant regarding same. The present study revealed that all aqueous and methanolic extracts of various parts *I. heterantha* contain many phytocompounds as depicted by preliminary phytochemical screening. The in-vitro antibacterial activity shows root, bark, leaves, and flower possess antibacterial activity. Methanolic root and bark have prominent activity on all strains while aqueous flower, root and leaves shows prominent activity on *Staphylococcus aureus*. In-vitro methanolic root, bark, and leaves have prominent antifungal activity while methanolic flower, aqueous root, bark, leaves and flower does not possess any antifungal activity. Since the adult Indian earth worm *Pheretima posthuma* shares morphological and physiological similarities with human intestinal roundworm parasites, the anthelmintic activity was conducted on this species. All the extracts have shown anthelmintic activity except aqueous flower. Prominent activity was shown by aqueous extracts of root and leaves followed by methanolic extracts of root and flower. The observed antifungal, antibacterial and anthelmintic activity revealed that root, bark, leaves and flower of *I. heterantha* as a good source for treating helmintic and other microbial infections. *I. heterantha* can be investigated further for isolation of bioactive compounds and the activity of those can be correlated with these specific activities, besides the isolated molecules may also serve as lead molecules for the synthesis of novel compounds with above mentioned activities.

### Supplementary Information

**Additional file 1: Table S1.** Extraction of different parts of Indigofera heterantha; **Figure S1.** Parts of Indigofera heterantha; **Figure S2.** Representative agar-well diffusion assay plates showing the antibacterial activity against Bacillus subtalis using different concentrations of the plant extracts and the positive control; **Figure S3.** Representative agar-well diffusion assay plates showing the antibacterial activity against E. coli using different concentrations of the plant extracts and positive control; **Figure S4.** Representative agar-well diffusion assay plates showing the antibacterial activity against Proteus vulgaris using different concentrations of the plant extracts and the positive control; **Figure S5.** Representative agar-well diffusion assay plates showing the antibacterial activity against Pseudomonas aeruginosa using different concentrations of the plant extracts and a positive control; **Figure S6.** Representative agar-well diffusion assay plates showing the antibacterial activity against Staphylococcus aureus using different concentrations of the plant extracts and positive control; **Figure S7.** Representative agar-well diffusion assay plates showing the antibacterial activity against Klebsiella pneumoniae using different concentrations of the plant extracts and positive control; **Figure S8.** Representative agar-well diffusion assay plates showing the antifungal activity against Penicillium chrysogenum using different concentrations of the plant extracts and the positive control; **Figure S9.** Representative agar-well diffusion assay plates showing the antifungal activity against Aspergillus fumigatus using different concentrations of the plant extracts and the positive control; **Figure S10.** Representative agar-well diffusion assay plates showing the antifungal activity against Saccharomyces cerevisiae using different concentrations of the plant extracts and the positive control; **Figure** **S11.** Representative agar-well diffusion assay plates showing the antifungal activity against Candida albicans using different concentrations of the plant extracts and a positive control. **Figure S12.** Representative Agar-well diffusion assay plates showing the anti-helminthic activity against Pheretima posthuma using different concentrations of the plant extractives and positive control.

## Data Availability

All data generated or analyzed during this study are included in this article.

## References

[1] O’Neil J. Review on antibiotic resistance. Antimicrobial Resistance: tackling a crisis for the health and wealth of nations. Heal. Wealth Nations. 2014;1–20. https://amr-review.org/sites/default/files/AMR%20Review%20Paper%20-%20Tackling%20a%20crisis%20for%20the%20health%20and%20wealth%20of%20nations_1.pdf. Accessed 25 Sept 2023.

[2] Reardon S (2014). Antibiotic resistance sweeping developing world. Nat News.

[3] Atanasov AG, Waltenberger B, Pferschy-Wenzig EM, Linder T, Wawrosch C, Uhrin P, Temml V, Wang L, Schwaiger S, Heiss EH, Rollinger JM (2015). Discovery and resupply of pharmacologically active plant-derived natural products: a review. Biotechnol Adv.

[4] Benarba B, Pandiella A (2020). Medicinal plants as sources of active molecules against COVID-19. Front Pharmacol.

[5] Bachrach ZY (2012). Contribution of selected medicinal plants for cancer prevention and therapy. ACTA FACULTATIS MEDICAE NAISSENSIS.

[6] Mazurek B, Chmiel M, Górecka B (2017). Fatty acids analysis using gas chromatography-mass spectrometer detector (GC/MSD)-method validation based on berry seed extract samples. Food Anal Methods.

[7] Pan SY, Zhou SF, Gao SH, Yu ZL, Zhang SF, Tang MK, Sun JN, Ma DL, Han YF, Fong WF, Ko KM (2013). New perspectives on how to discover drugs from herbal medicines: CAM’s outstanding contribution to modern therapeutics. Evidence-Based Complement Altern Med.

[8] Ward PI (2008). Environ Health Perspect. J Cell Biol.

[9] Oteng Mintah S, Asafo-Agyei T, Archer MA, Atta-Adjei Junior P, Boamah D, Kumadoh D, et al. Medicinal Plants for Treatment of Prevalent Diseases [Internet]. Pharmacognosy - Medicinal Plants. IntechOpen; 2019. Available from: 10.5772/intechopen.82049.

[10] Konappa N, Udayashankar AC, Krishnamurthy S, Pradeep CK, Chowdappa S, Jogaiah S (2020). GC–MS analysis of phytoconstituents from Amomumnilgiricum and molecular docking interactions of bioactive serverogenin acetate with target proteins. Sci Rep.

[11] Süntar I (2019). Importance of ethnopharmacological studies in drug discovery: role of medicinal plants. Phytochem Rev.

[12] Muzaffar AR, Baba SA (2015). Traditional uses of medicinal plants in Kashmir a review. Res Reviews: J Biology.

[13] Itoo A, Malik J, Shrivastava PN, Ashock K (2016). Ethnomedicinal value of shrub flora of Dachigam National Park traditionally used for health care practices by the inhabitants of Kashmir. Ann Pharmacy Pharmaceut Sci.

[14] Hazrat A, Nisar M, Shah J, Ahmad S (2011). Ethnobotanical study of some elite plants belonging to Dir, Kohistan valley, Khyber Pukhtunkhwa, Pakistan. Pak J Bot.

[15] Mohammad SA, Arshad M, Qureshi R (2015). Ethnobotanical inventory and folk uses of indigenous plants from Pir Nasoora National Park, Azad Jammu and Kashmir. Asian Pac J Trop Biomed.

[16] Wagay NA (2014). Medicinal flora and ethno-botanical knowledge of Baramulla Tehsil in Jammu and Kashmir, India. Int J Adv Biotechnol Res.

[17] Uddin G, Rahman TU, Arfan M, Waliullah WL, Rauf A, Khan I, Mohammad G, Choudhary MI (2011). In-vitro pharmacological investigations of aerial parts of Indigofera Heterantha. J Med Plants Res.

[18] Rahman TU, Uddin G, Nisa RU (2015). Spectroscopic and density functional theory studies of 7-hydroxy-3’-methoxyisoflavone: a new isoflavone from the seeds of *Indigofera Heterantha* (Wall). Spectrochim Acta A Mol Biomol Spectrosc.

[19] Gerometta E, Grondin I, Smadja J, Frederich M, Gauvin-Bialecki A (2020). A review of traditional uses, phytochemistry and pharmacology of the genus Indigofera. J Ethnopharmacol.

[20] Rehman A, Malik A, Mehmood S, Jahan E, Ahmad H (2005). Phytochemical studies on Indigofera Hetrantha. J Chem Soc Pak.

[21] Pal Singh I, Bharate SB (2006). Phloroglucinol compounds of natural origin. Nat Prod Rep.

[22] Rahman UT, Zeb MA, Liaqat W, Sajid M (2018). Phytochemistry and Pharmacology of Genus Indigofera: a review. Rec Nat Prod.

[23] Mehmood S, Rahman A, Ahmad Z, Afza N, Malik A, Ahmad H, Choudhary M (2008). I. Monoterpene glycosides from *Indigofera Hetrantha*. Nat Prod Res.

[24] Kaushik NK, Guha R, Saravanabalaji S (2015). Antiviral activity of *Indigofera Heterantha* Wall. Ex Brandis against herpes Simplex Virus—type 2 (HSV-2). Int J Adv Res.

[25] Khan H, Tariq SA, Khan MA (2011). Biological and phytochemical studies on corms of *Colchicum Luteum* Baker. J Med Plants Res.

[26] Aziz-Ur-Rehman, Malik A, Riaz N, Ahmad H, Nawaz SA, Choudhary MI (2005). Lipoxygenase inhibiting constituents from *Indigofera Heterantha*. Chem Pharm Bull.

[27] Gamble JS. A Manual of Indian Timbers. Publisher: Bishen Singh Mahendra Pal Singh; 1972.

[28] Phillips R, Rix M. Shrubs. Publisher: Pan Books Year; 1989. 0-330-30258-2 description: excellent photographs and a terse description of 1,900 species and cultivars.

[29] Huxley A. The New RHS Dictionary of Gardening. 1992. Publisher MacMillan Press Year 1992 ISBN 0-333-47494-5.

[30] Bean W. Trees and shrubs hardy in Great Britain. Vol 1–4 and supplement. Publication author Bean. W. Publisher Murray Year; 1981.

[31] Mohiuddin I, Kumar TR, Zargar MI, Wani SUD, Mahdi WA, Alshehri S, Alam P, Shakeel F (2022). GC-MS analysis, phytochemical screening, and antibacterial activity of cerana indica Propolis from Kashmir Region. Separations.

[32] Nabi M, Zargar MI, Tabassum N, Ganai BA, Wani SUD, Alshehri S, Alam P, Shakeel F (2022). Phytochemical profiling and antibacterial activity of methanol Leaf Extract of *Skimmia Anquetilia*. Plants.

[33] Mujeeb F, Bajpai P, Pathak N (2014). Phytochemical evaluation, antimicrobial activity, and determination of bioactive components from leaves of Aegle marmelos. Biomed Res Int.

[34] Hossain MA, AL-Raqmi KA, AL-Mijizy ZH, Weli AM, Al-Riyami Q (2013). Study of total phenol, flavonoids contents and phytochemical screening of various leaves crude extracts of locally grown Thymus vulgaris. Asian Pac J Trop Biomed.

[35] Aboody MSA, Mickymaray S (2020). Anti-fungal efficacy and mechanisms of flavonoids. Antibiotics.

[36] Ali M, Manjula SN, Wani SUD, Parihar VK, Mruthunjaya KM, Madhunapantula SV (2021). Protective role of herbal formulation-divine noni against cisplatin-induced cytotoxicity in healthy cells by activating Nrf2 expression: an in-vivo and in-vitro approach Phytomed. Plus.

[37] Ali M, Wani SUD, Salahuddin M (2023). Recent advance of herbal medicines in cancer- a molecular approach. Heliyon.

[38] Chasoo IA, Wani SUDW, Raja WY, Bhat ZA (2023). Physicochemical characterization, phytochemical analysis, and pharmacological evaluation of *Sambucus Wightiana*. Arab J Chem.

[39] Partap S, Kumar A, Sharma NK, Jha K. Luffa cylindrica: an important medicinal plant. J Nat Product Plant Resour. 2012;2:127–134. https://www.doc-developpement-durable.org/file/Culture/Culture-plantes-alimentaires/FICHES_PLANTES/luffa/Luffa%20Cylindrica_An%20important%20medicinal%20plant.pdf. (Accessed 14 Nov 2023).

[40] Wulandari L, Retnaningtyas Y, Lukman H (2016). Analysis of flavonoid in medicinal plant extract using infrared spectroscopy and chemometrics. J Anal Methods Chem.

[41] Qadir S, Wani IH, Rafiq S, Ganie SA, Masood A, Hamid R (2013). Evaluation of antimicrobial activity of a lectin isolated and purified from Indigofera heterantha. Advances in Bioscience and Biotechnology.

[42] Aziz MA, Khan AH, Adnan M, Izatullah I (2017). Traditional uses of medicinal plants reported by the indigenous communities and local herbal practitioners of Bajaur Agency, Federally Administrated Tribal Areas, Pakistan. J Ethnopharmacol.

[43] Bhagavan NB, Arunachalam S, Dhasarathan P, Kannan ND (2013). Evaluation of anti inflammatory activity of *Indigofera aspalathoides* Vahl in Swiss albino mice. J Pharm Res.

[44] Palani S, Kumar R, Kumar B (2009). Effect of the ethanolic extract of *Indigofera Barberi* (L.) in acute acetaminophen induced nephrotoxic rats. New Biotechnol.

