# Supplementary material for: In-vitro evaluation of Indigofera heterantha extracts for antibacterial, antifungal and anthelmintic activities
Source: J Pharm Health Care Sci. 2024 Jan 24;10:7. doi: 10.1186/s40780-024-00328-y (PMC10809583; doi:10.1186/s40780-024-00328-y)
Supplement: Supplementary file 1 — Additional file 1: Table S1. Extraction of different parts of Indigofera heterantha; Figure S1. Parts of Indigofera heterantha; Figure S2. Representative agar-well diffusion assay plates showing the antibacterial activity against Bacillus subtalis using different concentrations of the plant extracts and the positive control; Figure S3. Representative agar-well diffusion assay plates showing the antibacterial activity against E. coli using different concentrations of the plant extracts and positive control; Figure S4. Representative agar-well diffusion assay plates showing the antibacterial activity against Proteus vulgaris using different concentrations of the plant extracts and the positive control; Figure S5. Representative agar-well diffusion assay plates showing the antibacterial activity against Pseudomonas aeruginosa using different concentrations of the plant extracts and a positive control; Figure S6. Representative agar-well diffusion assay plates showing the antibacterial activity against Staphylococcus aureus using different concentrations of the plant extracts and positive control; Figure S7. Representative agar-well diffusion assay plates showing the antibacterial activity against Klebsiella pneumoniae using different concentrations of the plant extracts and positive control; Figure S8. Representative agar-well diffusion assay plates showing the antifungal activity against Penicillium chrysogenum using different concentrations of the plant extracts and the positive control; Figure S9. Representative agar-well diffusion assay plates showing the antifungal activity against Aspergillus fumigatus using different concentrations of the plant extracts and the positive control; Figure S10. Representative agar-well diffusion assay plates showing the antifungal activity against Saccharomyces cerevisiae using different concentrations of the plant extracts and the positive control; Figure S11. Representative agar-well diffusion assay plates showing the antifungal a [file 40780_2024_328_MOESM1_ESM.docx]

***Supplementary Information***

**Plant Description**

**Binomial name:** *Indigofera heterantha Wall. Ex.*

**Scientific classification**

Kingdom Plantae, Angiosperms

Order Fabales

Family Fabaceae

Subfamily Faboideae

Genus Indigofera

Species *Indigofera heterantha*

**Synonyms**

Common name - Himalayan indigo

Urdu - Jangli mulethi

Kashmiri name - Keiche, Jand

**Fig S1:** Parts of *Indigofera heterantha*

**Parts investigated:** Roots, Bark, Leaves, and Flowers

**Table S1. Extraction of different parts of *Indigofera heterantha***

| **Plant part** | **Solvent** | **Theoretical yield** | **Practical yield** | **%yield** |
| --- | --- | --- | --- | --- |
| Leaves | Methanol  Water | 600g  300g | 81.82g  50.33g | 13.64%  16.77% |
| Roots | Methanol  Water | 600g  300g | 76.36g  46g | 12.73%  15.33% |
| Bark | Methanol  Water | 300g  200g | 31.22g  25.33g | 10.41%  12.66% |
| Flower | Methanol  Water | 300g  300g | 22.45g  27.66g | 7.48%  9.22% |

**
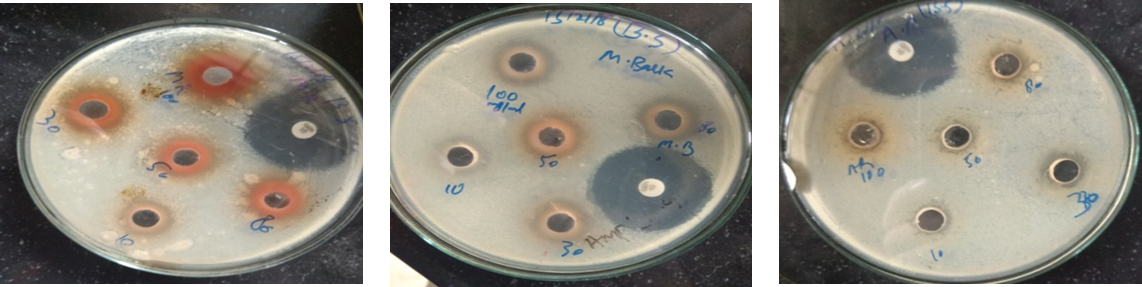
**

**Figure S2.** Representative Agar-well diffusion assay plates showing the anti-bacterial activity against *Bacillus subtalis* using different concentrations of the plant extractives and positive control.

**
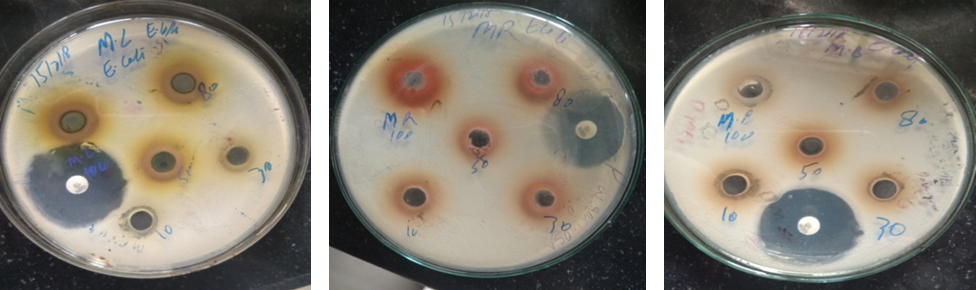
**

**Figure S3.** Representative Agar-well diffusion assay plates showing the anti-bacterial activity against *E. coli* using different concentrations of the plant extractives and positive control.

**
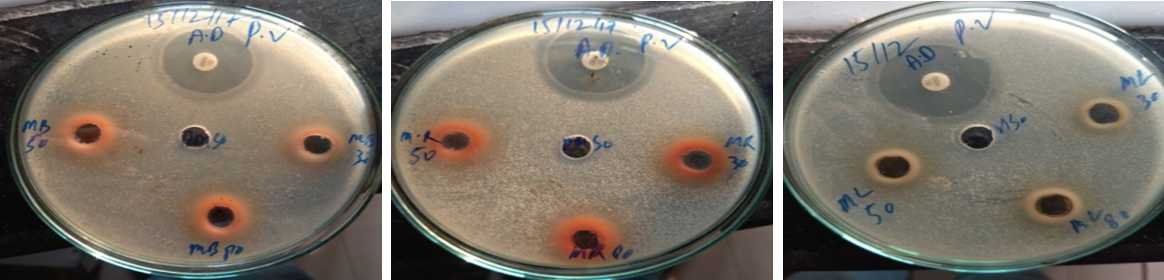
**

**Figure S4.** Representative Agar-well diffusion assay plates showing the anti-bacterial activity against *Proteus vulgaris* using different concentrations of the plant extractives and positive control.

**
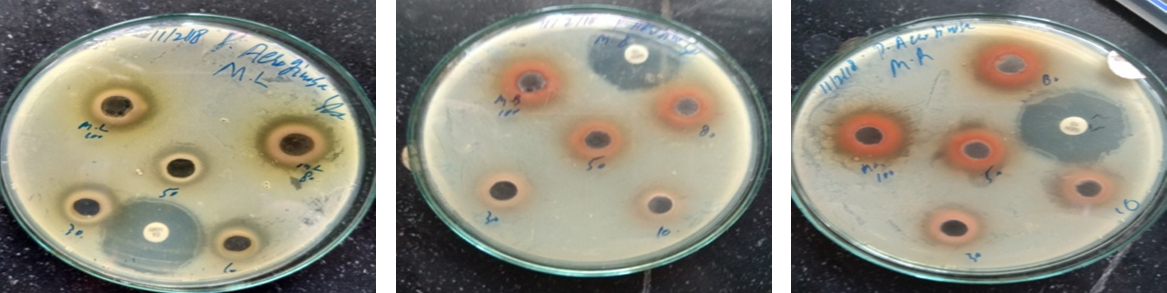
**

**Figure S5.** Representative Agar-well diffusion assay plates showing the anti-bacterial activity against *Pseudomonas Aeruginosa* using different concentrations of the plant extractives and positive control.

**
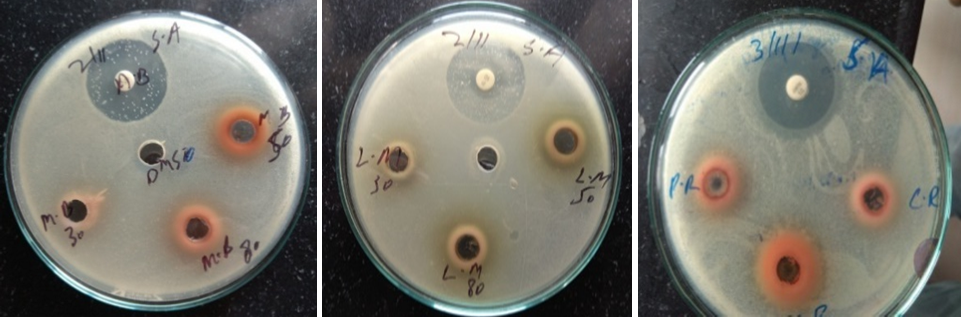
**

**Figure S6.** Representative Agar-well diffusion assay plates showing the anti-bacterial activity against *Staphylococcus aureus* using different concentrations of the plant extractives and positive control.


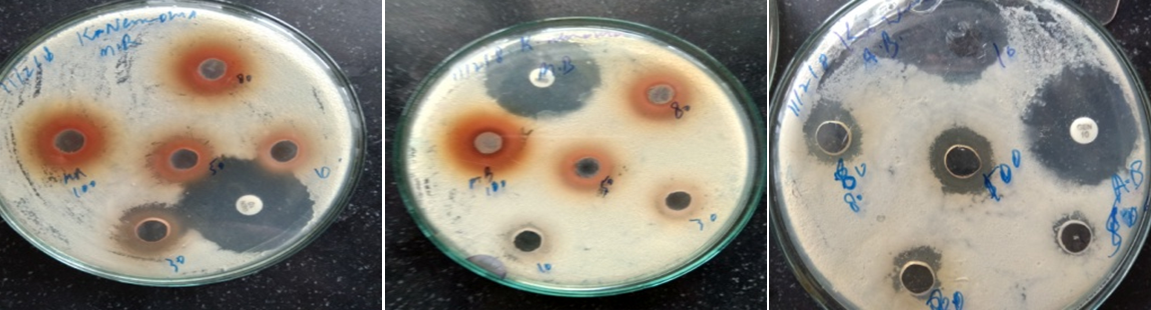


**Figure S7.** Representative Agar-well diffusion assay plates showing the anti-bacterial activity against *Klebsiella Pneumoniae* using different concentrations of the plant extractives and positive control.

| 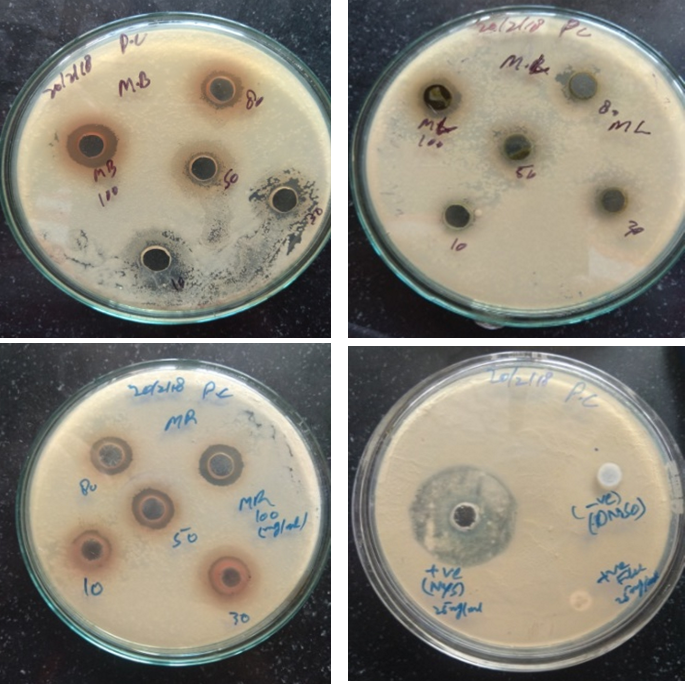 |  |
| --- | --- |

**Figure S8.** Representative Agar-well diffusion assay plates showing the anti-fungal activity against *Penicillium chrysogenum* using different concentrations of the plant extractives and positive control.

**
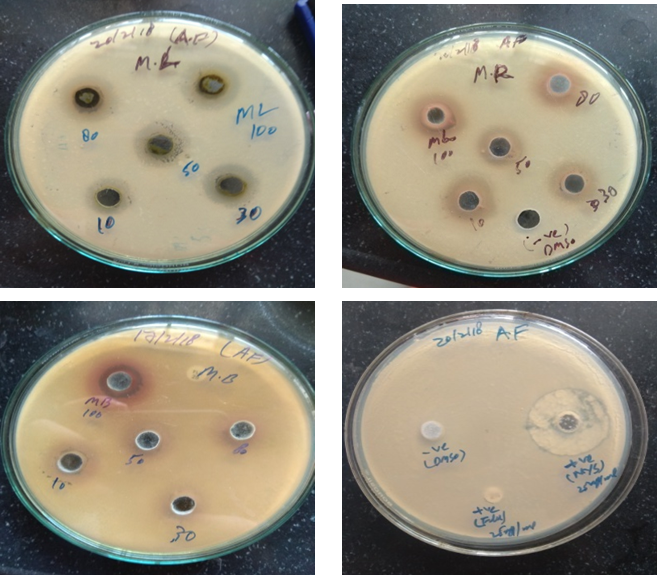
**

**Figure S9.** Representative Agar-well diffusion assay plates showing the anti-fungal activity against *Aspergillus Fumigatus* using different concentrations of the plant extractives and positive control.

**
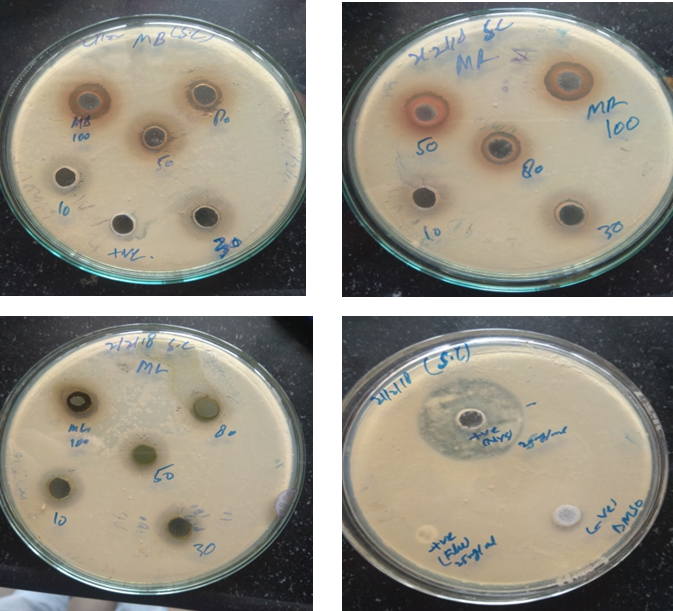
**

**Figure S10.** Representative Agar-well diffusion assay plates showing the anti-fungal activity against *Saccharomyces Cerevisiae* using different concentrations of the plant extractives and positive control.

**
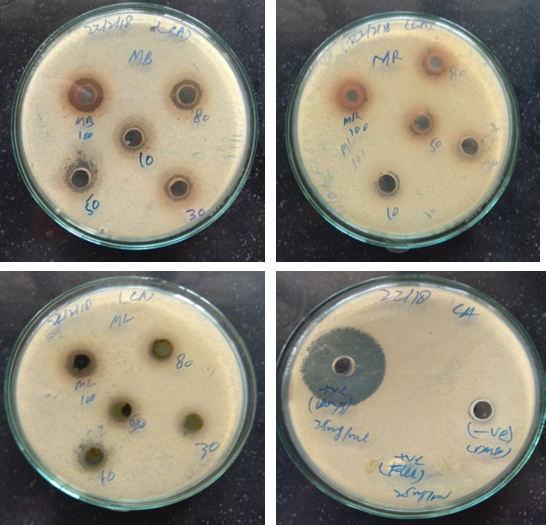
**

**Figure S11.** Representative Agar-well diffusion assay plates showing the anti-fungal activity against *Candida Albicans* using different concentrations of the plant extractives and positive control.


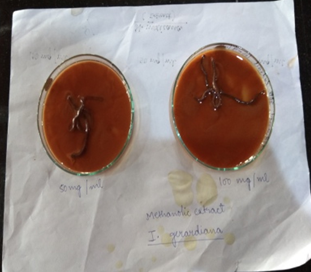

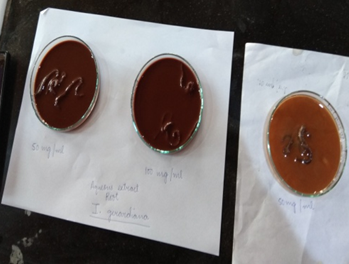


**Figure S12.** Representative Agar-well diffusion assay plates showing the anti-helminthic activity against *Pheretima posthuma* using different concentrations of the plant extractives and positive control.
